# Profiling of Volatile Organic Compounds, Including Halogenated Substances, in Okinawan Red Alga *Portieria hornemannii*

**DOI:** 10.3390/molecules30122534

**Published:** 2025-06-10

**Authors:** Kazuki Tani, Yu Sasaki, Takahiro Ishii, Yonathan Asikin

**Affiliations:** 1 Department of Bioscience and Biotechnology, Faculty of Agriculture, University of the Ryukyus, 1 Senbaru, Nishihara, Okinawa 903-0213, Japan; 2 The United Graduate School of Agricultural Sciences, Kagoshima University, 1-21-24 Korimoto, Kagoshima 890-0065, Japan

**Keywords:** volatile organic compounds, *Portieria hornemannii*, extraction temperature, solid-liquid extraction, halogenated compounds

## Abstract

The exploitation of underutilised resources is critical for achieving a sustainable society, and non-edible seaweeds are promising candidates. This study focused on the red alga *Portieria hornemannii* from Okinawa, Japan, a seaweed with a distinctive aroma, and determined its volatile organic compounds (VOCs) and halogenated secondary metabolites using headspace solid-phase microextraction gas chromatography–mass spectrometry (HS-SPME-GC-MS) at various extraction temperatures. HS-SPME-GC-MS analysis revealed 52 VOCs in Okinawan *P. hornemannii*, including predominant compounds α-pinenyl bromide (IUPAC name: 2-bromomethyl-6,6-dimethylbicyclo [3.1.1]hept-2-ene; halogenated monoterpene), myrcene disulfide (3-(6-methyl-2-methylidenehept-5-enylidene)dithiirane), and 5,6-dimethyl-1*H*-benzimidazole, the content of which in the extract increased with increasing extraction temperature from 30 to 60 °C. On the other hand, the β-myrcene (7-methyl-3-methyleneocta-1,6-diene) content, which likely contributes majorly to the distinct fresh odour of the algae, declined as the temperature increased. Furthermore, the proportion of β-myrcene obtained using SPME was significantly higher than that extracted using solvent liquid extraction (SLE) (7.20% in SPME at 30 °C vs. 0.09%, respectively). However, SLE-GC-MS provided a different *P. hornemannii* volatile profile, allowing for the acquisition of more furan-, alcohol-, ester-, and carboxylic acid-containing compounds. These data provide valuable information, such as a systematic analytical framework for volatiles profiling in the marine macroalgae *P. hornemannii*, with potential applicability in the development of food and fragrance products.

## 1. Introduction

Preserving the Earth’s environment during the Anthropocene is one of the key pillars of sustainable management conditions outlined in the Sustainable Development Goals agenda, which was adopted by the United Nations General Assembly in September 2015 [1,2]. According to this international framework, countries and regions are compelled to devise and implement specific measures to achieve a sustainable society. In particular, the effective utilisation of marine resources is a critical challenge in reconciling environmental protection with economic development. Among marine resources, seaweeds, which contribute significantly to the environment, economy, and society, have attracted increasing attention in recent years. Seaweeds comprise a diverse group of macroscopic, eukaryotic, and photosynthetic marine organisms with 10,000 different species being described to date [3]. Based on pigmentation, seaweeds are classified into three taxa: brown seaweed (Phaeophyceae), green seaweed (Chlorophyceae), and red seaweed (Rhodophyta) [4]. Seaweeds are abundant in vitamins, minerals, dietary fibres, carbohydrates, essential amino acids, proteins, fatty acids, and carotenoids and are widely consumed in Asian countries as ingredients in fresh, dried, or processed foods [5]. In recent years, research on the effective utilization of underexploited seaweed resources has attracted significant attention, with promising potential as novel food ingredients. For example, the red algae *Portieria hornemannii*, which is rich in dietary fibre and minerals, and is currently being considered for inclusion in cookies as nutritional support [6]. Furthermore, the nutritional components of this red alga may contribute to health promotion and disease prevention, thereby enhancing food functionality. The primary distinguishing characteristic of *P. hornemannii* is its peculiar scent; however, studies focusing on its aroma compounds are limited [7,8].

Volatile organic compounds (VOCs) are organic compounds that readily volatilise and exert biological and environmental effects. VOCs are emitted by diverse organisms including microorganisms, terrestrial plants, and marine organisms. In marine ecosystems, VOCs are recognised as secondary metabolites produced by seaweeds, corals, and bacteria and can be categorised into various groups, such as terpenes, halogenated compounds, sulphur compounds, and aldehydes [9,10]. Seaweeds are a major source of VOCs, exhibiting substantial variability in compound type and concentration depending on the species, developmental stage, and environmental conditions. VOCs are used in various industries, including biofuels, pharmaceuticals, food flavouring, and cosmetics [11,12]. Notably, red algae (Rhodophyta) produce significant quantities of halogenated monoterpenes, such as brominated and chlorinated terpenes, which are believed to function as a chemical deterrent [13,14]. Recently, these compounds have garnered attention owing to their potent biological activities, which make them promising candidates for drug discovery and pharmaceutical applications.

Our research group investigated Okinawan *P*. *hornemannii* and identified several unique secondary metabolites in its tissue, including one novel polyhalogenated cyclic monoterpenoid, 2(*R*)-chloro-1,6(*S*)-dibromo-3(8)(*Z*)-ochtoden-4(*R*)-ol, and several known non-volatile compounds [15]. These secondary metabolites may have potential applications in food. Although several non-volatile components have been identified, no systematic study has been conducted to characterise the VOCs emitted by *P*. *hornemannii*. Given the increased interest in marine-derived natural products for functional food and fragrance applications, determining the VOCs profile of this red alga is crucial for understanding its potential sensory and bioactivity properties. The current study attempts to address this knowledge gap by providing the first comprehensive analysis of VOCs in *P*. *hornemannii*. Therefore, the objective of the present study was to evaluate the volatile composition of Okinawan *P*. *hornemannii* using gas chromatography–mass spectrometry (GC-MS) with a particular focus on comparing the volatile profiles obtained via headspace solid-phase microextraction (HS-SPME) and solid–liquid extraction (SLE). This combined approach enabled us to confirm the identity of the various VOCs in *P*. *hornemannii*. The annotated VOCs were reported for the first time in the red alga *P*. *hornemannii*; however, their aroma properties remain unknown. Therefore, the current study provided a basis for further research on their potential applications in food flavouring and fragrance developments.

## 2. Results and Discussion

### 2.1. Effect of HS-SPME Temperature on the VOCs of P. hornemannii

**Figure 1:**
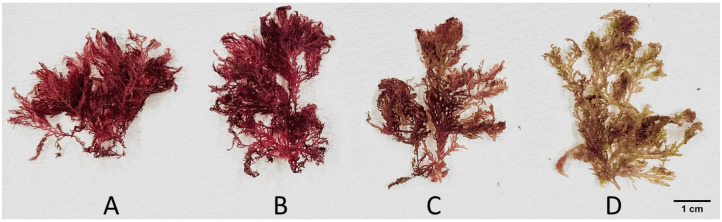
Variation in *P*. *hornemannii* colour extracted at: (**A**) 30 °C, (**B**) 45 °C, (**C**) 60 °C, and (**D**) 80 °C.

Treatment temperature is an important factor affecting the volatile composition of a material. In this study, we used divinylbenzene/carboxen/polydimethylsiloxane-coated fibres to examine the effects of various heating temperatures on the chemical constituents of Okinawan *P. hornemannii*. Heating at 80 °C, which is a common extraction temperature in food analysis [16], caused a colour change in the algal thallus from red to brown (a* colour space [green-to-red index]: from 53.5 to −2.6; b* colour space [blue-to-yellow index]: from 21.6 to 50.7) (Figure 1). Red algae may lose the colouration intensity of their brown pigments during high-temperature treatment, allowing the green hue of chlorophyll to become more prominent [17]. Furthermore, the characteristic aroma of fresh *P. hornemannii*, which was the focus of this study, disappeared under this extraction condition. Therefore, extraction at 80 °C was deemed unsuitable for obtaining the volatile constituents of *P. hornemannii*.

**Figure 2:**
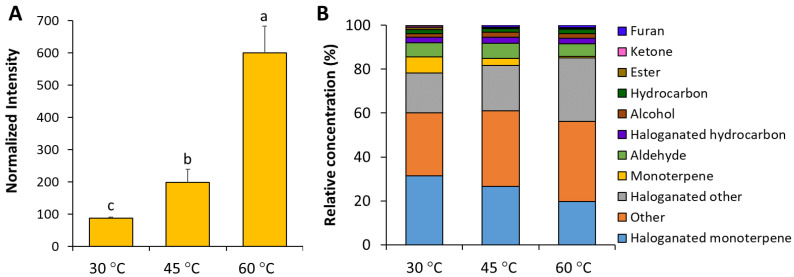
(**A**) Total normalised intensity and (**B**) relative concentration of VOCs in *P. hornemannii* obtained by HS-SPME-GC-MS analysis at varying extraction temperatures. Mean values of six replicates indicated by distinct letters are significantly different at *p* < 0.05.

**Figure 3:**
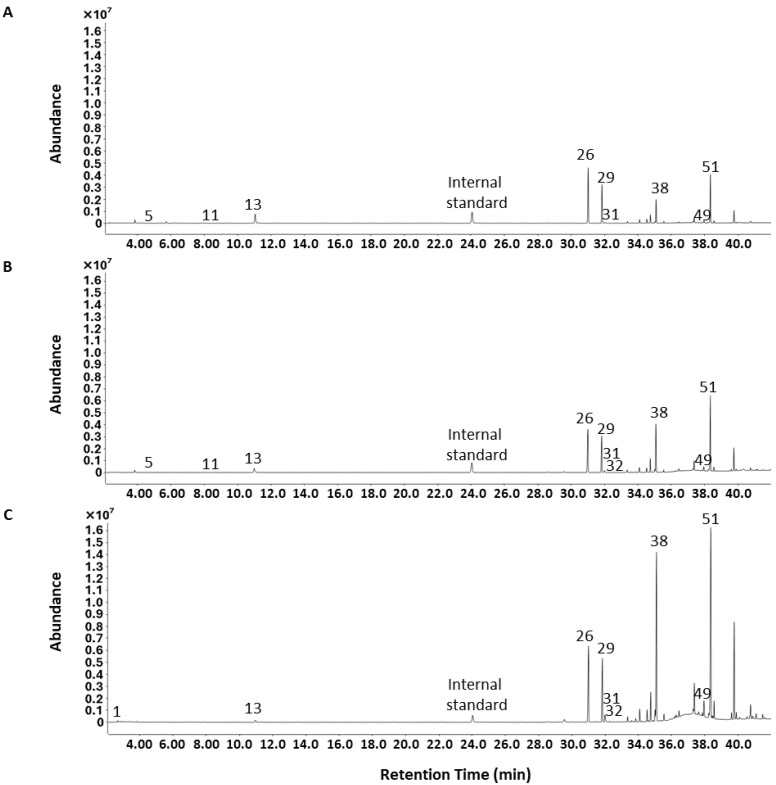
Representative chromatograms of VOCs of *P. hornemannii* obtained using HS-SPME-GC-MS analysis and varying extraction temperatures: (**A**) 30 °C, (**B**) 45 °C, and (**C**) 60 °C. The peak numbers refer to the compounds in Table 1; internal standard (cyclohexanol).

The effect of extraction temperature on the volatile composition of *P. hornemannii* was further examined by heating at 30, 45, and 60 °C, whereupon the colour of the red alga in general remained unchanged (Figure 1). The HS-SPME-GC-MS analysis showed that the highest total amount of components was obtained at an extraction temperature of 60 °C, followed by that at 45 and 30 °C, with significant differences being observed between each condition (*p* < 0.05) (Figure 2A). Higher temperatures appeared to increase the volatility of the chemical components of *P. hornemannii* that could be absorbed by the SPME fibre, as shown by the higher total ion intensity in the respective chromatograms (Figure 3). Higher temperature extraction at 45 and 60 °C resulted in larger peak areas of volatile compounds that were eluted in the last part of the chromatogram. This increase can be attributed to the adsorption of less volatile compounds and altered non-native substances of *P. hornemannii* onto the fibre coating at higher temperatures. The analysis revealed 11 chemical classes of VOCs, including monoterpenes, halogenated monoterpenes, halogenated hydrocarbons, other halogenated compounds, alcohols, aldehydes, esters, furans, hydrocarbons, and ketones (Figure 2B). As the temperature increased, the relative percentage of VOCs known to contribute to the fresh aroma characteristics of various marine resources and foods, including monoterpenes and halogenated monoterpenes [11,12], decreased from 7.44% to 0.63% and from 31.34% to 19.76%, respectively, whereas the proportion of VOCs that did not match any chemical class in the MS libraries (classified as other compounds) tended to increase.

A total of 132 chromatographic peaks were detected (Figure 3), wherein 52 VOCs were annotated in the extract of Okinawan *P. hornemannii*, with the major components being α-pinenyl bromide (IUPAC name: 2-bromomethyl-6,6-dimethylbicyclo [3.1.1]hept-2-ene), myrcene disulfide (3-(6-methyl-2-methylidenehept-5-enylidene)dithiirane), 5,6-dimethyl-1*H*-benzimidazole, 1,3-dibromoadamantane, and *N*-(2-bromophenyl)hex-5-enamide (Table 1 and Supplementary Table S1). Among unknown components, 2 moderate and 48 minor peaks were detected in the extracts, respectively. In addition, these halogenated compounds have also been reported in red algae of different origins and thus are natural compounds [6,7,8,9,10]. The major VOCs of *P. hornemannii* are rich in halogenated compounds, differing from the VOC profiles of other seaweed species previously reported [18,19,20]. The Okinawan red alga contained a high proportion of the monoterpene β-myrcene (7-methyl-3-methyleneocta-1,6-diene; odour threshold (OT) value, 0.042 mg/kg), which is characterised by a spicy and woody aroma [21,22]; thus, this volatile compound likely contributes substantially to its distinctive aroma. However, as the extraction temperature increased, the amount of β-myrcene significantly decreased, evidenced by the decrease in the corresponding normalised intensity from 6.29 (relative concentration, 7.22%) at 30 °C to 6.54 (3.29%) at 45 °C and 3.16 (0.53%) at 60 °C. The decrease in β-myrcene at higher temperatures may be due to either its thermal breakdown or a reduction in its ability to adsorb onto the SPME fibre coating [12]. Furthermore, elevated temperatures also altered the contents of halogenated monoterpenes of *P. hornemannii*, including α-pinenyl bromide, 2-bromo-*p*-cymene (2-bromo-1-methyl-4-propan-2-ylbenzene), and 1,3-dibromoadamantane. For instance, a significant 3.5-fold increase in the normalised intensity was observed for α-pinenyl bromide (normalised intensity of 25.20 at 30 °C vs. 87.52 at 60 °C). The presence of these halogenated compounds highlights the distinctiveness of the biochemical properties of *P. hornemannii* compared with other seaweed species. Moreover, it confirms its ability to produce abundant halogenated compounds, particularly halogenated monoterpenes, a trait that may be attributed to environmental adaptations or evolutionary factors [18,23,24]. Additionally, temperature-dependent behaviour was observed for other predominant compounds, including myrcene disulfide and 5,6-dimethyl-1*H*-benzimidazole. The evolution in the normalised intensity corresponding to these compounds was as follows: myrcene disulfide increased by 3.8-fold (15.01 at 30 °C vs. 57.36 at 60 °C) and 5,6-dimethyl-1*H*-benzimidazole increased by 14.7-fold (8.85 at 30 °C vs. 129.95 at 60 °C). This result implies that elevated temperatures promote the release of these relatively low-volatility compounds [12]. On the other hand, hydrocarbons, such as 1,3-octadiene and 1-ethylcyclohexa-1,4-diene were detected in extracts obtained at 30 and 45 °C but were not detected in those at 60 °C. In contrast, acetaldehyde (ethanal; OT value, 1.3 mg/kg) [22] and 4-ethenyl-1,2-dimethylbenzene were exclusively detected at high temperatures. These findings clearly demonstrate that extraction temperature has a significant influence on the compositional profile of the detected volatiles. Statistical analysis revealed significant differences (*p* < 0.05) between the extraction temperatures for numerous compounds. Specifically, while the content of a number of monoterpenes, including β-myrcene, did not differ significantly between 30 and 45 °C, significant differences were observed between that at the lower temperatures and at 60 °C. This result suggests that heating at lower temperature conditions, and in particular at 30 °C, are best suited for the extraction of VOCs from *P. hornemannii*.

**Figure 4:**
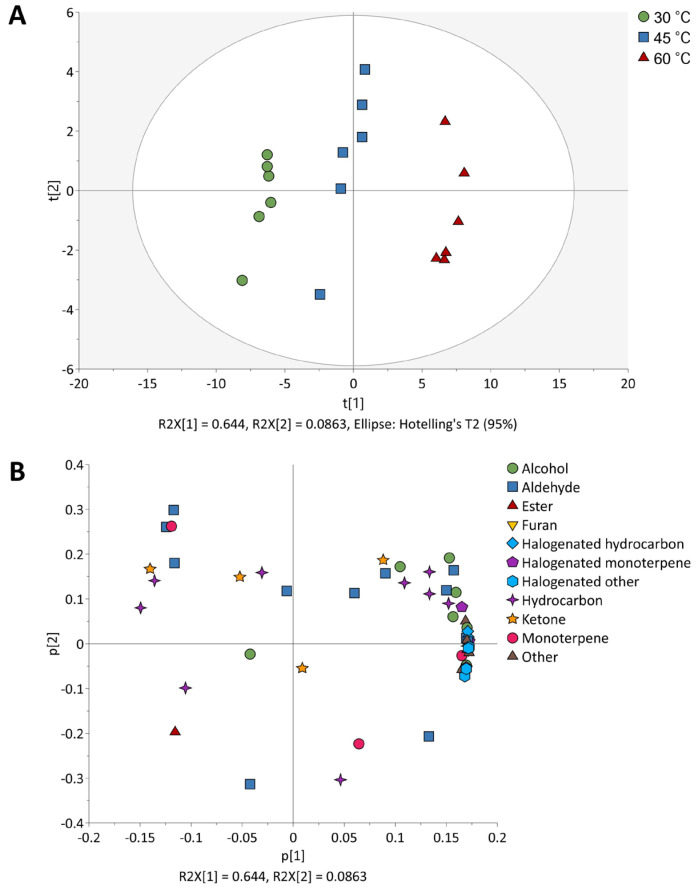
(**A**) PCA score plots and (**B**) factor loadings of VOCs in *P. hornemannii* obtained using HS-SPME-GC-MS analysis and varying extraction temperatures.

Variations in the VOCs obtained at differing extraction temperatures were further examined through multivariate analysis using primary component analysis (PCA) (Figure 4). The first two principal components (PCs) of the VOCs in *P*. *hornemannii* obtained at differing extraction temperatures explained 73.0% of the total variance, accounted for by PC1 and PC2 (64.4 and 8.6%, respectively). The score plots for each extraction temperature were closely clustered, indicating that heating at the same temperature consistently affected the VOCs profile of *P*. *hornemannii* (Figure 4A). The PCA score plot, based on the normalised intensities of VOCs in the red alga, exhibited variations across all extraction temperatures and allowed for clear differentiation among them. A factor-loading plot was constructed to examine the relationship between the score plots and each compound. The monoterpenes, with the exception of β-myrcene, and most halogenated monoterpenes and halogenated hydrocarbons were distributed along the positive direction of PC1 (Figure 4B). Additionally, aldehydes, esters, and several hydrocarbons were placed on the negative side of PC1. Accordingly, the replicate results for extraction at 30 °C, which were plotted negatively on PC1, tended to indicate higher levels of β-myrcene and certain aldehydes and hydrocarbons compared to that at other extraction temperatures. In contrast, the replicates of extraction at 60 °C, which were plotted positively on PC1, tended to show higher intensities of alcohols, halogenated compounds, and other substances. Additionally, the replicates at median temperature (45 °C) were positioned between these two extremes, indicating that the extraction temperature exerted a significant influence on the detected compounds. The PCA visualisation plot thus confirmed that extraction temperature alters the VOCs profile of *P*. *hornemannii*, whereby β-myrcene as an aroma-valuable monoterpene was effectively preserved at lower temperatures (Figure 4 and Table 1). Preserving such monoterpenes during heat-mediated extraction is important because they are considered key aromatic substances in marine resources including algae owing to their distinct natural fresh scents with potential applicability [25].

The VOCs profile of Okinawan *P. hornemannii* identified herein indicated its distinctiveness by revealing a unique distribution of components compared with those of previously reported algae [18,19,20]. Such differences can be attributed to chemical diversity among genera, environmental influences, and genetic factors. Therefore, these results represent valuable data for seaweed metabolite research, offering new insights for potential applications. The influence of the extraction temperature on the detection of monoterpenes and halogenated monoterpenes has been previously reported [26]. The results of this study provide valuable empirical data that support these findings. By leveraging the identified temperature-dependent emission characteristics, the selective release of monoterpenes with aroma properties and halogenated monoterpenes with potent antimicrobial activity can be achieved under appropriate thermal conditions. The approach has potential applicability in the food industry, specifically for the efficient obtainment of aroma enhancers and functional additives.

### 2.2. Identification of VOCs Emitted from P. hornemannii Using SLE-GC-MS

**Figure 5:**
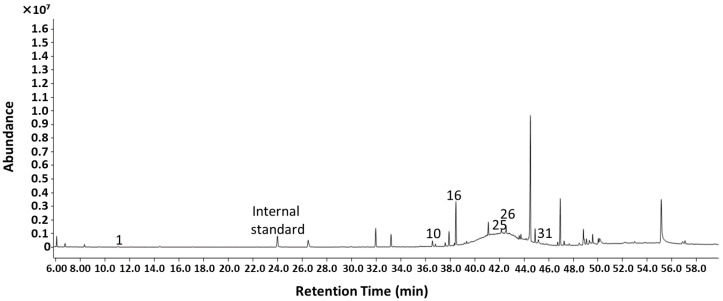
Representative chromatograms of VOCs of *P. hornemannii* obtained using SLE-GC-MS analysis. The peak numbers refer to the compounds in Table 2; internal standard (cyclohexanol).

A total of 91 chromatographic peaks were detected (Figure 5), wherein 50 VOCs comprising 12 chemical classes were annotated using SLE-GC-MS (Table 2). Among unknown components, 20 moderate and 21 minor peaks were detected in the extract, respectively. Substances containing furan, alcohol, hydrocarbon, and ester functionalities were predominant in the extract, being highly compatible with the solvent used in SLE. This volatile composition differed from that obtained using HS-SPME-GC-MS (Table 1 and Table 2). Namely, the major constituents of the SLE extract were 2-ethyl-1-benzofuran, 4′-(β-chloroethyl) acetophenone (IUPAC name: 1-[4-(2-chloroethyl)phenyl]ethenone), and neophytadiene (7,11,15-trimethyl-3-methylidenehexadec-1-ene), accounting for 10.65%, 9.54%, and 8.93%, respectively. Moreover, the SLE extract contained furans, alcohols, esters, and carboxylic acids that were not detected by SPME. Furthermore, the β-myrcene (7-methyl-3-methyleneocta-1,6-diene) amount obtained via SLE was considerably lower than that via SPME (0.09% vs. 7.20, extracted at 30 °C). However, this is consistent with the findings of previous studies [6,7], describing that β-myrcene was detected in trace levels in the SLE extracts of red algae. Only 11 compounds were detected by both SLE-GC-MS and SPME-GC-MS techniques, with SPME consistently affording significantly higher concentrations, exhibiting concentration differentials ranging from 4-fold to as much as 80-fold (Table 1). Abundant amounts of monoterpenes and halogenated compounds were detected by SPME-GC-MS, but were significantly lower in the SLE extract. Significant differences were noted for the shared compounds between the two extraction techniques (*p* < 0.05), with the exception of (2*E*)-octa-2,7-dien-1-ol and 5,7-dimethyl-1*H*-indazole, the proportions of which were comparable among the extraction methods employed. Each chemical group potentially contributed distinct odour characteristics to the overall aroma profile of *P*. *hornemannii*. For example, monoterpenes, exemplified by β-myrcene, typically impart citrusy or herbaceous notes [27], whereas halogenated compounds frequently contribute medicinal olfactory properties [28]. Carboxylic acids and esters commonly contribute to fruity aromatic notes and fatty or waxy qualities, whereas furans and ketones typically impart sweet, caramel-like, or nutty olfactory properties to the comprehensive aroma profile [29]. These findings underscore the significant impact of targeted extraction methodologies and solvent selection on the VOCs profile and suggest that HS-SPME-GC-MS is more suitable for analysing the distinctive aroma components of *P*. *hornemannii*.

SLE-GC-MS analysis provided lower levels of monoterpenes and halogenated monoterpenes than HS-SPME-GC-MS. Notably, this suggests that gas-phase extraction is more suitable for halogenated monoterpenes than high-polarity solvents. This finding underscores the importance of selecting an appropriate extraction technique for the accurate characterisation of the VOCs profile of marine natural products and their precise analysis. The results of this study provide fundamental data for defining the production profile and functional significance of halogenated monoterpenes, thereby serving as a crucial foundation for future applications. Further research into the usage of different extraction techniques is warranted. In particular, the use of alternative methods such as distillation or mechanical pressing should also be explored and compared with the present findings to obtain a better understanding of the VOC profiles in *P*. *hornemannii*. Additionally, to assess the olfactory relevance of the identified volatile compounds, future studies should incorporate the calculation of odour activity values, which consider both compound concentrations and their OT values. This approach will allow for a more accurate and comprehensive evaluation of the contribution of individual volatiles to the overall aroma profile.

## 3. Materials and Methods

### 3.1. Materials

*P*. *hornemannii* was harvested from White Beach, Uruma City, Okinawa, Japan, during April 2024. The specimens were thoroughly rinsed three times with distilled water and dried using a towel paper. The colour traits of the tissue were assessed using ImageJ (Ver. 1.54i 03, National Institutes of Health, Bethesda, MD, USA), and its L*a*b* colour spaces were subsequently generated by https://colorizer.org/ (assessed on 25 May 2024). Subsequently, the dried specimens were cryopulverised using a multi-bead shocker at 1500 rpm for 20 s (Yasui Kikai, Osaka, Japan) and the obtained powder was stored at −80 °C prior to use. The powder was acclimated to room temperature (25 °C) prior to volatile extraction.

### 3.2. VOCs Extraction Using HS-SPME

SPME fibre (divinylbenzene/carboxen/polydimethylsiloxane 50/30 μm, Supelco, Bellefonte, PA, USA) used in HS-SPME was preconditioned at 270 °C for 30 min. Briefly, *P*. *hornemannii* powder (100 mg) and cyclohexanol (20 µL, 50 µg/mL) as an internal standard (Fujifilm Wako Pure Chemical, Osaka, Japan) were dispensed into a 20 mL headspace vial (GL Sciences, Tokyo, Japan). The vial was then incubated for 30 min at 30, 45, 60, or 80 °C in a CombiPAL autosampler (CTC Analytics, Zwingen, Switzerland) to extract the volatiles. The VOCs were desorbed from the SPME fibre into the GC-MS inlet for 2 min. All extractions were performed in triplicate.

### 3.3. SLE Extraction

Briefly, *P*. *hornemannii* powder (300 mg) was spiked with 50 µL of the internal standard cyclohexanol (50 µg/mL, Fujifilm Wako Pure Chemical). The powder was then placed in 10 mL of ethyl acetate (Fujifilm Wako Pure Chemical) [7,8] and the mixture was allowed to stand in the dark at 28 °C for 6 h. Subsequently, the mixture was dehydrated using 1 g of anhydrous sodium sulphate at 4 °C for 12 h. The extract was then concentrated to 150 µL under a gentle nitrogen stream using a Kuderna-Danish concentrator. The concentrated extract (1 µL) was then injected into the GC-MS. All extractions were performed in triplicate.

### 3.4. Gas Chromatography-Mass Spectrometry (GC-MS) Analysis

GC-MS analysis was performed using an Agilent 7890 B GC (Agilent Technologies, Santa Clara, CA, USA) coupled with a 5977A MS. Helium was used as the carrier gas at a flow rate of 1 mL/min. The GC inlet (desorption temperature) was set to 250 °C with a split ratio of 10:1. The volatiles were separated using a DB-Wax or DB-5MS capillary column (30 m × 0.25 mm, 0.25 μm, Agilent Technologies). The oven temperature for SPME injection was initially set to 50 °C for 2 min, then increased to 100 °C at 2 °C/min, to 230 °C at 10 °C/min, and finally held at 230 °C for 2 min. The oven temperature for SLE extraction was initially set to 50 °C for 2 min, increased to 100 °C at 2 °C/min, followed by an increase to 230 °C at 5 °C/min, and finally held at 230 °C for 7 min. MS acquisition was performed for *m*/*z* 35–350 in electron ionisation mode (70 eV). The ion source and transfer line temperatures were both maintained at 230 °C. The VOCs were identified by comparing the obtained GC-MS data with those in the NIST Version 17 and Wiley 9th libraries (similarity > 80%) and linear retention index (LRI) comparisons (<|20|) of *n*-alkane (C_7_–C_30_) measurements in capillary columns. Normalised peak intensities were calibrated to the peak area response of the internal standard.

### 3.5. Statistical Analysis

Statistical differences between the mean values of the groups were analysed using Tukey’s multiple comparison test or unpaired Tukey’s test (for two groups) (GraphPad Prism Version 9; GraphPad Software, Boston, MA, USA). Data for PCA were normalised using log_10_-transformation and unit variance scaling and visualised using SIMCA Version 17 (Sartorius, Göttingen, Germany).

## 4. Conclusions

The volatile composition of the Okinawan red alga *P. hornemannii* was found to differ significantly depending on the extraction temperature. VOCs extraction using SPME led to distinct VOCs profiles when conducted at 30, 45, and 60 °C. Lower extraction temperatures tended to preserve monoterpenes and halogenated monoterpenes. The trend was also observed in β-myrcene (7-methyl-3-methyleneocta-1,6-diene), an important aroma-imparting terpene in *P*. *hornemannii*. These results demonstrate that extraction temperature plays a critical role in VOCs detection, with heating at 30 °C and the use of SPME being the ideal conditions for VOCs extraction from this red alga. A different volatile composition of *P*. *hornemannii* was observed using SLE with ethyl acetate, wherein a greater proportion of furan-, alcohol-, ester-, and carboxylic acid-containing compounds and a much lower relative concentration of monoterpenes and halogenated monoterpenes were observed. The contrast in the volatile profiles demonstrates that the non-destructive SPME technique is sensitive to aroma-important substances such as monoterpenes, whereas the SLE method affords VOCs that are compatible with the extraction solvent. In addition, monoterpenes and halogenated monoterpenes were detected at lower levels when SLE-GC-MS was applied compared with that using HS-SPME-GC-MS, suggesting that halogenated compounds are more suitable for gas-phase extraction. This highlights the importance of selecting an appropriate extraction technique to accurately characterise the VOCs profile of natural marine products. The present study provides a guideline for evaluating the volatile composition of *P*. *hornemannii* via the two extraction methods, which may be further utilised in various industries, such as the food, fragrance, and cosmetic sectors. The two extraction methods were compared to reveal their advantages and potential applications. The SPME technique, being solvent-free, cost-effective, and environmentally sustainable, offers an efficient extraction method for profiling thermally labile and aroma-relevant volatiles such as monoterpenes and halogenated monoterpenes. In contrast, the SLE method, which uses organic solvents, enables the extraction of a broader range of semi-volatile and polar compounds, including alcohols, esters, and carboxylic acids, which may be valuable in developing applications for the food, fragrance, and cosmetic industries. Thus, the selection of an appropriate extraction strategy should be guided by the specific analytical or industrial objectives, particularly when targeting functional volatile components from marine natural resources such as *P. hornemannii*. Additionally, the results of this study provide valuable primary information for further research on VOCs from *P. hornemannii* populations distributed across Japan, offering deeper insights into how environmental factors affect the chemical diversity of red algae.

## Figures and Tables

**Table 1:** Normalised intensities of VOCs in *P. hornemannii* obtained using HS-SPME-GC-MS analysis and varying extraction temperatures.

No.	RI (DB-WAX) ^1^	RI (DB-5MS) ^2^	Compound (Written in IUPAC Name)	Chemical Group	30 °C	45 °C	60 °C
1	711	-	Ethanal	Aldehyde	nd	nd	0.43 ± 0.44
2	783	-	Propanal	Aldehyde	0.06 ± 0.03 b	0.10 ± 0.06 b	0.31 ± 0.22 a
3	877	-	2-Methyl-2-propenal	Aldehyde	0.05 ± 0.03 ab	0.06 ± 0.03 a	0.01 ± 0.02 b
4	884	608	Ethyl acetate	Ester	0.93 ± 0.21 a	0.80 ± 0.23 a	0.54 ± 0.19 b
5	949	-	Octa-1,3-diene	Hydrocarbon	0.05 ± 0.01 a	0.05 ± 0.04 a	nd
6	976	699	Pentanal	Aldehyde	0.15 ± 0.04 a	0.12 ± 0.03 a	0.07 ± 0.01 b
7	1018	681	Pent-1-en-3-one	Ketone	0.29 ± 0.03 a	0.21 ± 0.03 ab	0.16 ± 0.03 b
8	1029	-	(3*E*,6*E*)-Octa-1,3,6-triene	Monoterpene	0.11 ± 0.03 a	0.11 ± 0.03 a	0.14 ± 0.03 a
9	1051	694	Pentane-2,3-dione	Ketone	0.04 ± 0.01 a	0.05 ± 0.01 a	0.08 ± 0.06 a
10	1077	800	Hexanal	Aldehyde	0.35 ± 0.03 a	0.33 ± 0.06 a	0.22 ± 0.05 b
11	1100	-	1-Ethylcyclohexa-1,4-diene	Hydrocarbon	0.05 ± 0.01 a	0.04 ± 0.02 a	nd
12	1124	747	*(E)*-Pent-2-enal	Aldehyde	0.11 ± 0.02 a	0.17 ± 0.04 b	0.24 ± 0.05 c
13	1157	988	7-Methyl-3-methyleneocta-1,6-diene	Monoterpene	6.29 ± 0.89 a	6.54 ± 1.52 a	3.16 ± 0.70 b
14	1191	-	3-Butyl-4-ethenylcyclopentene	Hydrocarbon	0.17 ± 0.06 a	0.11 ± 0.10 a	0.04 ± 0.06 b
15	1213	847	*(E)*-Hex-2-enal	Aldehyde	0.07 ± 0.02 a	0.13 ± 0.06 a	0.13 ± 0.09 a
16	1282	-	Cyclohexanone	Ketone	0.12 ± 0.01 a	0.13 ± 0.05 a	0.09 ± 0.06 a
17	1297	-	Oct-1-yn-3-ol	Alcohol	0.14 ± 0.02 b	0.25 ± 0.08 a	0.33 ± 0.04 a
18	1320	762	*(Z)*-Pent-2-en-1-ol	Alcohol	0.08 ± 0.01 b	0.13 ± 0.05 ab	0.16 ± 0.05 a
19	1349	1175	6-Butylcyclohepta-1,4-diene	Hydrocarbon	0.12 ± 0.03 b	0.16 ± 0.05 ab	0.22 ± 0.05 a
20	1369	1156	6-[(*Z*)-But-1-enyl]cyclohepta-1,4-diene	Hydrocarbon	0.40 ± 0.05 b	0.49 ± 0.10 ab	0.62 ± 0.13 a
21	1424	1055	*(E)*-Oct-2-enal	Aldehyde	0.05 ± 0.03 c	0.10 ± 0.02 b	0.22 ± 0.02 a
22	1458	977	Oct-1-en-3-ol	Alcohol	0.09 ± 0.01 c	0.21 ± 0.05 b	0.26 ± 0.02 a
23	1489	970	(5*Z*)-Octa-1,5-dien-3-ol	Alcohol	0.21 ± 0.03 c	0.58 ± 0.13 b	0.71 ± 0.05 a
24	1500	-	[(*E*)-But-1-enyl]benzene	Hydrocarbon	0.02 ± 0.01 c	0.09 ± 0.03 b	0.28 ± 0.04 a
25	1514	958	Benzenecarbaldehyde	Aldehyde	1.32 ± 0.77 b	2.76 ± 1.61 b	7.44 ± 2.87 a
26	1566	1258	2-Bromomethyl-6,6-dimethylbicyclo [3.1.1]hept-2-ene	Halogenated monoterpene	25.20 ± 2.00 c	47.22 ± 14.86 b	87.52 ± 17.98 a
27	1579	-	1-(Methoxymethoxymethyl)-4-prop-1-en-2-ylcyclohexene	Monoterpene	0.05 ± 0.01 b	0.12 ± 0.03 b	0.32 ± 0.10 a
28	1583	1158	(2*E*,6*Z*)-Nona-2,6-dienal	Aldehyde	0.09 ± 0.02 b	0.17 ± 0.04 b	0.54 ± 0.10 a
29	1596	1280	3-(6-Methyl-2-methylidenehept-5-enylidene)dithiirane	Other	15.01 ± 0.76 c	30.64 ± 7.66 b	57.36 ± 8.55 a
30	1602	1101	2-Methyl-1-benzofuran	Furan	0.41 ± 0.02 c	1.85 ± 0.33 b	7.22 ± 0.82 a
31	1618	1270	2-Bromo-1-methyl-4-propan-2-ylbenzene	Halogenated monoterpene	0.10 ± 0.01 c	0.27 ± 0.06 b	0.88 ± 0.14 a
32	1643	-	4-Ethenyl-1,2-dimethylbenzene	Hydrocarbon	nd	0.07 ± 0.06 b	0.25 ± 0.06 a
33	1673	1284	2-Bromoadamantane	Halogenated hydrocarbon	0.72 ± 0.04 c	1.78 ± 0.39 b	4.09 ± 0.53 a
34	1686	1067	(2*E*)-Octa-2,7-dien-1-ol	Alcohol	0.14 ± 0.02 c	0.46 ± 0.10 b	1.06 ± 0.12 a
35	1698	-	Heptadecane	Hydrocarbon	0.10 ± 0.03 c	0.51 ± 0.12 b	2.12 ± 0.37 a
36	1718	1356	4-Chlorooctahydro-2,4-methano-indene	Halogenated hydrocarbon	1.50 ± 0.10 c	4.00 ± 1.09 b	10.37 ± 1.88 a
37	1757	1198	4-(1-Methylethyl)benzaldehyde	Aldehyde	3.06 ± 0.26 c	8.58 ± 1.85 b	21.31 ± 4.30 a
38	1777	1239	5,6-Dimethyl-1*H*-benzimidazole	Other	8.85 ± 0.35 c	31.53 ± 6.42 b	129.95 ± 18.26 a
39	1811	1352	4-(4-Methylpent-3-enyl)-3,6-dihydrodithiine	Other	0.61 ± 0.08 c	1.63 ± 0.38 b	5.51 ± 0.62 a
40	1881	-	2,7-Dimethylocta-2,6-dien-1-ol	Alcohol	0.52 ± 0.04 c	2.40 ± 0.21 b	9.83 ± 0.73 a
41	1903	1227	5,7-Dimethyl-1*H*-indazole	Other	0.23 ± 0.04 b	2.08 ± 1.06 a	10.50 ± 3.71 a
42	1915	-	Pentamethylbenzenesulphonamide	Other	0.40 ± 0.18 b	2.23 ± 1.08 a	15.99 ± 4.32 a
43	1926	-	[(*E*)-Pent-2-en-2-yl]benzene	Hydrocarbon	0.19 ± 0.25 b	0.69 ± 0.83 b	5.81 ± 3.10 a
44	1927	-	1-Methyl-1,3-dihydroinden-2-one	Ketone	0.06 ± 0.09 a	0.08 ± 0.21 a	2.28 ± 3.54 a
45	1935	-	2,2-Dimethyl-1,3-dihydroindene	Hydrocarbon	0.47 ± 0.12 b	1.09 ± 0.83 a	3.55 ± 2.85 a
46	1943	-	(*E*)-3-(4-Methylphenyl)prop-2-enal	Aldehyde	0.37 ± 0.05 a	0.79 ± 0.63 a	3.89 ± 5.04 a
47	1953	1476	1-Acetyl-3-chloro-adamantane	Halogenated other	0.67 ± 0.08 b	2.45 ± 0.78 b	13.51 ± 2.22 a
48	1975	-	2-Buta-1,3-dien-2-ylphenol	Alcohol	0.14 ± 0.18 a	0.31 ± 0.41 a	0.42 ± 1.03 a
49	2005	-	1,3-Dibromoadamantane	Halogenated monoterpene	2.02 ± 0.18 b	5.33 ± 1.13 b	30.38 ± 5.17 a
50	2037	1375	2,4,6-Trimethylbenzoyl chloride	Halogenated other	0.43 ± 0.05 c	1.66 ± 0.36 b	6.68 ± 0.63 a
51	2046	1541	*N*-(2-Bromophenyl)hex-5-enamide	Halogenated other	14.39 ± 2.24 c	36.36 ± 4.44 b	150.94 ± 20.41 a
52	2054	-	6,6-Dimethylbicyclo [3.1.1]hept-2-ene-2-carbonyl bromide	Halogenated other	0.13 ± 0.04 c	0.50 ± 0.11 b	2.93 ± 0.20 a

Each value is expressed as the mean ± standard deviation of six replicates; nd = not detected. Means in the same row followed by different letters are significantly different at *p* < 0.05. ^1^ Retention indices relative to *n*-alkanes on a DB-Wax column (used for compound identification and value quantification). ^2^ Retention indices relative to *n*-alkanes on a D5-MS column (used for compound identification).

**Table 2:** Relative concentration (%) of VOCs obtained from *P. hornemannii* using SLE-GC-MS analysis, compared to HS-SPME-GC-MS with extraction at 30 °C.

No	RI (DB-Wax)	Compound (Written in IUPAC Name)	Chemical Group	SLE (%)	SPME 30 °C (%)
1	1160	7-Methyl-3-methyleneocta-1,6-diene	Monoterpene	0.09 ± 0.04 b	7.20 ± 0.85 a
2	1233	Acetic anhydride	Other	0.15 ± 0.04	nd
3	1448	Acetic acid	Carboxylic acid	1.60 ± 0.97	nd
4	1513	Benzenecarbaldehyde	Aldehyde	0.09 ± 0.05 b	1.51 ± 0.88 a
5	1563	2-Bromomethyl-6,6-dimethylbicyclo [3.1.1]hept-2-ene	Halogenated monoterpene	2.94 ± 1.11 b	28.88 ± 1.58 a
6	1600	2-Methyl-1-benzofuran	Furan	0.21 ± 0.04 b	0.47 ± 0.02 a
7	1622	2-(2-Ethoxyethoxy)ethanol	Alcohol	0.08 ± 0.06	nd
8	1635	2-Hydroxyethyl acetate	Ester	0.04 ± 0.02	nd
9	1668	2-Bromoadamantane	Halogenated hydrocarbon	0.19 ± 0.10 b	0.83 ± 0.03 a
10	1686	(2*E*)-Octa-2,7-dien-1-ol	Alcohol	0.13 ± 0.08 a	0.16 ± 0.03 a
11	1700	Heptadecane	Hydrocarbon	1.61 ± 0.36 a	0.11 ± 0.03 b
12	1709	(1*E*,4*E*,8*E*)-2,6,6,9-Tetramethylcycloundeca-1,4,8-triene	Hydrocarbon	0.51 ± 0.09 b	0.71 ± 0.09 a
13	1743	2,6-Diethylbromobenzene	Halogenated hydrocarbon	0.16 ± 0.07	nd
14	1750	4-(1-Methylethyl)benzaldehyde	Aldehyde	2.92 ± 0.26 b	3.51 ± 0.32 a
15	1766	5,6-Dimethyl-1*H*-benzimidazole	Other	0.79 ± 0.12	nd
16	1771	2-Ethyl-1-benzofuran	Furan	10.65 ± 0.87	nd
17	1797	2-(2-Butoxyethoxy)ethanol	Alcohol	1.63 ± 0.75	nd
18	1851	2-Methyl-2,3-dihydroinden-1-one	Ketone	4.99 ± 2.67	nd
19	1853	2,3-Dimethyl-1-benzofuran	Furan	1.12 ± 0.68	nd
20	1857	2,3-Dihydro-3-methyl-1*H*-inden-1-one	Ketone	2.92 ± 0.83	nd
21	1894	1-Methyl-1,3-dihydroinden-2-one	Ketone	1.28 ± 1.04	nd
22	1896	2-Ethenyl-1,3,5-trimethylbenzene	Hydrocarbon	2.62 ± 1.28	nd
23	1915	Ethyl-2-benzofuran	Furan	2.46 ± 1.58	nd
24	1925	7,11,15-Trimethyl-3-methylidenehexadec-1-ene	Hydrocarbon	8.93 ± 1.70	nd
25	1943	1-[4-(2-Chloroethyl)phenyl]ethanone	Halogenated other	9.54 ± 2.05	nd
26	1955	[(2*E*,7*R*,11*R*)-3,7,11,15-Tetramethylhexadec-2-enyl]acetate	Ester	5.92 ± 1.14	nd
27	1976	6-Methoxy-3-methylbenzofuran	Furan	3.76 ± 1.87	nd
28	1992	2-*tert*-Butylphenol mesylate	Other	4.07 ± 0.40	nd
29	2054	*N*-(2-Bromophenyl)hex-5-enamide	Halogenated other	4.30 ± 0.50 b	16.54 ± 2.80 a
30	2067	5,7-Dimethyl-1*H*-indazole	Other	1.57 ± 2.44 a	0.27 ± 0.04 a
31	2086	1-Bromo-2,2-dimethyl-1-(1-hexynyl)-cyclopropane	Halogenated hydrocarbon	1.12 ± 0.51	nd
32	2096	2,4,7,9-Tetramethyl-5-decyne-4,7-diol	Alcohol	0.83 ± 0.28	nd
33	2147	2,4,6-Trimethylbenzoyl chloride	Halogenated other	0.77 ± 0.12 a	0.49 ± 0.05 b
34	2186	1-(3-Chloro-2-propenyl)-4-methoxy-benzene	Halogenated hydrocarbon	0.27 ± 0.06	nd
35	2221	Methyl 14-methylpentadecanoate	Ester	0.12 ± 0.07	nd
36	2225	1-Acetyl-5-chloro-adamantane	Halogenated other	0.01 ± 0.01	nd
37	2258	7,7-Bis(bromomethyl)-1-methylbicyclo [2.2.1]heptan-2-one	Halogenated other	3.92 ± 0.77	nd
38	2271	9,10-Dichloro-tricyclo [3.3.2.0(1,5)]decan-2-one	Halogenated other	1.91 ± 0.28	nd
39	2285	(*Z*)-1-Bromo-8-methyl-hydrindan-2-on	Halogenated other	2.01 ± 0.57	nd
40	2311	2,4-di-*tert*-Butylphenol	Alcohol	0.44 ± 0.38	nd
41	2361	Docosahexaenoic acid	Carboxylic acid	0.83 ± 0.20	nd
42	2388	Methyl docosahexaenoate	Ester	0.81 ± 0.30	nd
43	2449	*(Z)*-9-Octadecenoic acid	Carboxylic acid	1.01 ± 0.70	nd
44	2485	Dodecanoic acid	Carboxylic acid	1.09 ± 0.44	nd
45	2541	4-Benzyloxytricyclo [4.3.1.0(3,8)]decan-10-ol	Alcohol	0.89 ± 0.36	nd
46	2548	Bis-(2-methylpropyl)ester-1,2-benzenedicarboxylic acid	Ester	0.82 ± 0.42	nd
47	2593	2-Octadecyloxyethanol	Alcohol	0.18 ± 0.18	nd
48	2599	Docosane	Hydrocarbon	1.03 ± 0.68	nd
49	2666	Hexadecyl-2-ethylhexanoate	Ester	0.38 ± 0.61	nd
50	2719	Octan-2-yl palmitate	Ester	0.24 ± 0.24	nd

Each value is expressed as the mean ± standard deviation of six replicates; nd = not detected. Means in the same row followed by different letters are significantly different at *p* < 0.05.

## Data Availability

The raw data supporting the conclusions of this article will be made available by the authors on request.

## Supplementary Materials

The following supporting information can be downloaded at: https://www.mdpi.com/article/10.3390/molecules30122534/s1, Supplementary Table S1. Retention indices (RI) of VOCs detected in *P. hornemannii* by HS-SPME-GC-MS and their corresponding literature RI values.
