# Supplementary material for: Profiling of Volatile Organic Compounds, Including Halogenated Substances, in Okinawan Red Alga Portieria hornemannii
Source: Molecules. 2025 Jun 10;30(12):2534. doi: 10.3390/molecules30122534 (PMC12195807; doi:10.3390/molecules30122534)
Supplement: Supplementary file 1 [file molecules-30-02534-s001.zip › molecules-3582143-supplementary.pdf]

Table S1. Retention indices (RI) of VOCs detected in *P. hornemannii* by HS-SPME-GC-MS and their corresponding literature RI values.

No.	Calculated RI (DB-WAX)	Literature RI (DB-WAX)	Calculated RI (DB5-MS)	Literature RI (DB-5MS)	Compound (written in IUPAC name)
1	711	713	-	381	Ethanal
2	783	784	-	511	Propanal
3	877	883	-	507	2-Methyl-2-propenal
4	884	884	608	612	Ethyl acetate
5	949	954	-	827	Octa-1,3-diene
6	976	975	699	698	Pentanal
7	1018	1019	681	680	Pent-1-en-3-one
8	1029	1048	-	-	(3*E*,6*E*)-Octa-1,3,6-triene
9	1051	1050	694	696	Pentane-2,3-dione
10	1077	1077	800	801	Hexanal
11	1100	-	-	-	1-Ethylcyclohexa-1,4-diene
12	1124	1124	747	759	*(E)*-Pent-2-enal
13	1157	1157	988	989	7-Methyl-3-methyleneocta-1,6-diene
14	1191	-	-	-	3-Butyl-4-ethenylcyclopentene
15	1213	1212	847	854	*(E)*-Hex-2-enal
16	1282	1281	-	875	Cyclohexanone
17	1297	-	-	-	Oct-1-yn-3-ol
18	1320	1322	762	763	*(Z)*-Pent-2-en-1-ol
19	1349	-	1175	-	6-Butylcyclohepta-1,4-diene
20	1369	-	1156	-	6-[(*Z*)-But-1-enyl]cyclohepta-1,4-diene
21	1424	1425	1055	1056	*(E)*-Oct-2-enal
22	1458	1458	977	977	Oct-1-en-3-ol
23	1489	-	970	974	(5*Z*)-Octa-1,5-dien-3-ol
24	1500	-	-	-	[(*E*)-But-1-enyl]benzene
25	1514	1513	958	960	Benzenecarbaldehyde
26	1566	-	1258	-	2-Bromomethyl-6,6-dimethylbicyclo[3.1.1]hept-2-ene
27	1579	-	-	-	1-(Methoxymethoxymethyl)-4-prop-1-en-2-ylcyclohexene
28	1583	1583	1158	1156	(2*E*,6*Z*)-Nona-2,6-dienal

Table S1. Retention indices (RI) of VOCs detected in *P. hornemannii* by HS-SPME-GC-MS and their corresponding literature RI values (continued).

No.	Calculated RI (DB-WAX)	Literature RI (DB-WAX)	Calculated RI (DB5-MS)	Literature RI (DB-5MS)	Compound (written in IUPAC name)
29	1596	-	1280	-	3-(6-Methyl-2-methylidenehept-5-enylidene)dithiirane
30	1602	-	1101	-	2-Methyl-1-benzofuran
31	1618	-	1270	-	2-Bromo-1-methyl-4-propan-2-ylbenzene
32	1643	-	-	-	4-Ethenyl-1,2-dimethylbenzene
33	1673	-	1284	-	2-Bromoadamantane
34	1686	-	1067	-	(2*E*)-Octa-2,7-dien-1-ol
35	1698	1700	-	287	Heptadecane
36	1718	-	1356	-	4-Chlorooctahydro-2,4-methano-indene
37	1757	1760	1198	1189	4-(1-Methylethyl)benzaldehyde
38	1777	-	1239	-	5,6-Dimethyl-1*H*-benzimidazole
39	1811	-	1352	-	4-(4-Methylpent-3-enyl)-3,6-dihydrodithiine
40	1881	-	-	-	2,7-Dimethylocta-2,6-dien-1-ol
41	1903	-	1227	-	5,7-Dimethyl-1*H*-indazole
42	1915	-	-	-	Pentamethylbenzenesulphonamide
43	1926	-	-	-	[(*E*)-Pent-2-en-2-yl]benzene
44	1927	-	-	-	1-Methyl-1,3-dihydroinden-2-one
45	1935	-	-	-	2,2-Dimethyl-1,3-dihydroindene
46	1943	-	-	-	(*E*)-3-(4-Methylphenyl)prop-2-enal
47	1953	-	1476	-	1-Acetyl-3-chloro-adamantane
48	1975	-	-	-	2-Buta-1,3-dien-2-ylphenol
49	2005	-	-	-	1,3-Dibromoadamantane
50	2037	-	1375	-	2,4,6-Trimethylbenzoyl chloride
51	2046	-	1541	-	*N*-(2-Bromophenyl)hex-5-enamide
52	2054	-	-	-	6,6-Dimethylbicyclo[3.1.1]hept-2-ene-2-carbonyl bromide
